# A Narrative Review of Tuberculosis and Diabetes Mellitus: The Double Burden Epidemiology, the Current Moment and the Future

**DOI:** 10.7759/cureus.96016

**Published:** 2025-11-03

**Authors:** Emmanuel M Sindato

**Affiliations:** 1 Department of Internal Medicine, School of Medicine and Dentistry, The University of Dodoma, Dodoma, TZA

**Keywords:** diabetes mellitus, double burden phenomenon, epidemiology, integration, low and middle-income countries, narrative review, tuberculosis

## Abstract

Tuberculosis and diabetes are major global health concerns, with their prevalences projected to increase disproportionately in the low- and middle-income countries (LMICs). The convergence of tuberculosis and diabetes impact is also expected to rise, posing a negative synergism that complicates disease management and outcomes. This review aims to summarise tuberculosis and diabetes double burden epidemiology, the overlap, the current moment and future prospects, including exploration of cost-effective interventions to enhance tuberculosis and diabetes integrated care. A literature search was conducted in PubMed, Embase, African Journals Online and Google Scholar on January 1, 2025, to identify studies published between January 1, 2020, and December 1, 2024, on the double burden of tuberculosis and diabetes, as well as integration models. There were no language restrictions. The diagnostic criteria for tuberculosis and diabetes, as well as the integration models, were aligned with WHO guidelines. After screening abstracts and full texts, 35 articles were included in this review. The double burden of tuberculosis and diabetes presents unique challenges at present, but unlocking potential for new opportunities to transform global healthcare delivery amid the increasing double burden disease phenomenon. Integrating tuberculosis and diabetes care is essential to avoid further strain on the already fragile healthcare delivery systems in LMICs. Integrated care enhances bi-directional screening, promotes a holistic approach of treatment and management, and optimises outcomes for both diseases.

## Introduction and background

Globally, tuberculosis remains a substantial public health concern, which is highly prevalent in resource-constrained settings and regions with a high HIV burden. Concurrently, for the past six decades, low- and middle-income countries (LMICs) have undergone an epidemiological transition with steadily rising prevalence of non-communicable diseases (NCDs), including diabetes. The increasing NCD burden in LMICs creates a double burden disease phenomenon in these regions [1,2]. Diabetes is associated with an increased risk of poor tuberculosis treatment outcomes and may increase the risk of developing primary multidrug-resistant tuberculosis [3-5]. Additionally, tuberculosis can unmask a diabetes diagnosis and can lead to poorly-controlled diabetes [6-8]. Furthermore, co-morbidity of tuberculosis and diabetes is associated with a two-fold increase in the risk of mortality during tuberculosis treatment, and a two-fold increase in the risk of tuberculosis relapse after treatment completion. Tuberculosis and diabetes convergence will continue to increase in LMICs due to the increasing burden of diabetes and factors that hinder global tuberculosis elimination goals, such as emerging epidemics, such as COVID-19, changes in geopolitical polices, and shifts in global health funding and aid. This review summarises the tuberculosis and diabetes epidemiology, overlap, the current moment and future prospects, including exploration of cost-effective interventions to enhance tuberculosis and diabetes integrated care.

## Review

Methods

A literature search was conducted in PubMed, Embase, African Journals Online and Google Scholar on 1 January 2025 to identify studies published between 1 January 2020 and 1 December 2024 on the double burden of tuberculosis and diabetes, as well as the integration models. There were no language restrictions. The diagnostic criteria for tuberculosis and diabetes, as well as the integration models, were aligned with WHO guidelines. After removing duplicates, screening abstracts and full texts, 35 articles were included in this review.

Burden of diabetes

Diabetes is highly prevalent throughout the world, and the burden of this disease is rising. Literature indicates that the number of individuals living with diabetes quadrupled from 200 million in 1990 to 830 million in 2022 [9]. During this period, the global prevalence of diabetes among adults aged 18 and above nearly doubled, rising from 6.8% in 1990 to 14.1% in 2022 [10-13]. Diabetes has increased dramatically in developing countries, particularly in Eastern Asia and sub-Saharan Africa, due to rapid ongoing urbanisation and advancement in the standard of living alongside adaptation of Western lifestyles [12,13].

Diabetes poses a range of serious and fatal complications, especially if undiagnosed or poorly-controlled state. The most pronounced complications include adult blindness, neuropathies, non-traumatic lower extremity amputation, kidney disease, stroke and cardiovascular disease [14]. Management of these complications has significant financial implications, with substantial negative impact on the economy in LMICs [15], the regions where only about 20% of the population have health insurance coverage [16].

Burden of tuberculosis

From the discovery of Mycobacterium tuberculosis (MTB) by Robert Koch in 1882 to the advent of multiple anti-TB drugs, the morbidity and mortality of tuberculosis declined significantly until the early 1980s, when tuberculosis began to increase alongside the HIV epidemic. To date, mortality due to tuberculosis, multidrug-resistant tuberculosis (MDR-TB) and extensively drug-resistant tuberculosis (XDR-TB) is the highest of all infectious agents globally [17]. Early identification and cure of tuberculosis is the highest priority in the context of reducing morbidity and mortality for infected individuals and preventing tuberculosis transmission.

According to the Global Tuberculosis Report 2024, yearly deaths caused by tuberculosis dropped between the years 2010 and 2019; however, the number increased in the years 2020 and 2021 due to disruptions to tuberculosis diagnosis and treatment during the COVID-19 pandemic. Tuberculosis mortality trend as per the WHO 2024 report shows that it has re-emerged as the leading infectious cause of death from a single agent, following a brief period, from 2020 through 2022, during which COVID-19 was the leading infectious cause of death [17]. Post-COVID-19 era tuberculosis resurgence as the leading infectious cause underscores the persistent global burden of tuberculosis and the need for sustained public health efforts to contain it.

The reported number of people newly diagnosed with tuberculosis fell from 7.1 million in 2019 to 5.8 million in 2020 and 6.4 million in 2021, translating into a significant increase in the number of people with undiagnosed and untreated tuberculosis [17]. In the year 2023, tuberculosis was responsible for approximately 1.09 million deaths globally among HIV-negative people, and an estimated 161,000 deaths among people with HIV [17,18]. Current published meta-analyses have shown that Asian and sub-Saharan African countries have a significant burden of latent tuberculosis infection, with prevalence of 21.0% and 22.4% respectively [19,20], which indicates a large population at risk of developing tuberculosis disease, especially in the presence of diabetes and HIV co-morbidities.

Tuberculosis and diabetes: the overlap

As diabetes becomes increasingly prevalent in developing regions, where tuberculosis is already prevalent, these two epidemics will continue to converge into the tuberculosis-diabetes double burden disease, with a negative synergism in health outcomes.

The prevalence of tuberculosis among patients with diabetes varies globally depending on region and population studied, tuberculosis case-finding strategy, and HIV burden. Epidemiological studies have shown that diabetes increases the relative risk for tuberculosis threefold [4], with larger effect estimates in regions of higher tuberculosis prevalence [4]. In studies from India and the USA/Mexico border, it was observed that 15-25% of tuberculosis cases were related to diabetes [4].

In addition to this convergence of the two epidemics, it is already recognised that there is significant overlap and interaction between tuberculosis and diabetes. Tuberculosis has been shown to increase the risk of developing diabetes in both adults and paediatric patients, and diabetes has been associated with an increasing prevalence of tuberculosis in comparison to the non-diabetic population. Furthermore, oral hypoglycaemic agents have been associated with resistance to rifampicin, and tuberculosis worsens diabetes and makes glycaemia control challenging [3,5].

Diabetes patients who are co-infected with tuberculosis may have atypical presentations. They may present with poor glycaemic control, dry cough without classical constitutional symptoms for tuberculosis, or atypical radiological findings. In poorly-controlled diabetes patients, tuberculosis should be suspected as an underlying factor, and in diabetes patients with cough, there should be a high index of suspicion for tuberculosis disease [21,22].

Most available information concerning the interaction between tuberculosis and diabetes comes from Western countries, even though Eastern Asia and sub-Saharan Africa. Africa has the highest prevalence of tuberculosis due to HIV co-infection. Further research regarding the tuberculosis-diabetes interaction in these regions is of prime importance to better understand the phenomenon and plan for strategies to combat this problem.

Pharmacological aspects of diabetes and tuberculosis

There are various aspects to consider in the pharmacological management of tuberculosis and diabetes coexistence. Drug-drug interactions and adverse effects should be anticipated and addressed at the time drugs are initiated.

Rifampicin is a powerful inducer of cytochrome P450 system enzymes and phase II enzymes [23]. Induction of these enzymes can lead to accelerated metabolism of drugs given with rifampicin and reduced treatment effect. The sulfonylureas are among the most commonly used oral hypoglycaemic drugs for patients with diabetes, and are substrates of cytochrome P450 isoenzyme 2C9 (CYP2C9). Pharmacokinetic studies show that serum concentrations of sulfonylureas are decreased by 34% when given simultaneously with rifampicin, leading to decreased effectiveness of sulfonylurea drugs in controlling blood glucose levels [23]. Additionally, diabetes is increasingly recognised as a significant risk factor for the development of MDR-TB due to suspected pharmacokinetic factors, among others. The current literature shows that the burden of MDR-TB among patients with diabetes is about 10-fold higher as compared to individuals without diabetes [5,17]. The mechanisms underlying this increased risk are multifactorial, encompassing impairment in the host immune response and altered pharmacokinetics of anti-tuberculosis drugs [5].

Predictors of diabetes among patients with tuberculosis

Studies have shown that various risk factors and predictors of glucose metabolism disorders exist. Generally, patients afflicted with tuberculosis face the same cardio-metabolic risks as the general population, such as older age, male sex, high body mass index (BMI), increased waist circumference, physical inactivity, excessive alcohol consumption, and family history of glucose metabolism disorders.

A study in Mwanza, Tanzania by Faurholt-Jepsen et al. found that additional predictors of diabetes among patients with tuberculosis include age between 45-55 years and being underweight (BMI < 16 kg/m2) [24]. Furthermore, Ledergerber et al. showed that HIV infection, particularly among patients with a low CD4 count and those on protease-inhibitor-based therapy, increases the risk of diabetes [25]. HIV is highly prevalent in LICMs regions, posing an increased risk in these regions. The presence of these risk factors among tuberculosis patients should prompt diabetes screening [22].

Radiological perspectives on tuberculosis diabetes comorbidity

Existing literature has suggested that pulmonary tuberculosis among patients with diabetes presents with more severe radiological findings than non-diabetic patients [26]. Furthermore, diabetes may contribute to atypical tuberculosis chest radiographic findings, with increased involvement of the lower lung fields as compared to the typical presentation of tuberculosis in the upper lobes [26,27]. The atypical presentation can lead to misdiagnosis of tuberculosis as pneumonia, carcinoma, or lung abscesses. Therefore, it is important to have a high index of suspicion for tuberculosis in the setting of an abnormal chest radiogram among individuals with diabetes [26,27].

The future

Amid the increasingly prevalent phenomenon of the tuberculosis-diabetes double burden disease, it is essential for healthcare facilities to integrate tuberculosis and diabetes clinics in order to provide holistic care within the context of the coexistence of NCDs and communicable diseases (CDs).

The integration of tuberculosis and diabetes clinics significantly improves patient care by enabling bidirectional screening of these diseases, leading to earlier diagnosis and better outcomes for both conditions [22]. This integration facilitates coordinated treatment and management by offering streamlined care with shared medical records, reduced patient burden from fewer appointments, improved medication adherence, and minimised drug interactions [22,28]. Patients also benefit from enhanced education and self-management support. A tuberculosis and diabetes silo clinic will further optimise healthcare workers' efficiency, particularly in LMICs, where human resources for the health workforce are substantially limited [29,30].

The INTE-AFRICA pragmatic cluster-randomised controlled trial provides a realistic understanding of the integrated care models of NCDs and CDs in sub-Saharan Africa. Key messages from the trial entail that the integration process of NCDs and CDs is feasible and acceptable to both patients and healthcare providers [31]. Furthermore, the trial indicates that when NCDs and CDs care is integrated, there are significant advancements in outcomes, including higher retention rates and improved cost effectiveness when compared to the stand-alone NCDs or CDs clinics in the region [31]. Therefore, it is important to adopt these lessons, engage with policymakers and prioritise scaling up the NCDs and CDs integrated care model.

Operationalising the integration of NCDs and CDs care entails leveraging existing infrastructure and available resources to provide optimal care for both NCDs and CDs without compromising care delivery of either type of disease. WHO has proposed a structural module for the integration process that focuses on screening strategies, preventive measures, early diagnosis, management and treatment outcomes of both tuberculosis and diabetes as one package [21].

Implementation of bi-directional screening of tuberculosis and diabetes is paramount to reduce the morbidity and mortality in the co-existence of these conditions. Bi-directional screening will enhance the WHO call for early tuberculosis case detection among people with diabetes and early diabetes diagnosis among people with tuberculosis [32]. Holistic management, that is, bidirectional screening, comprehensive treatment of both conditions, active prevention, health promotion, and training for healthcare workers should be employed whenever there is co-existence of tuberculosis and diabetes [21,22]. Currently, the WHO recommends the same tuberculosis treatment regimen to be prescribed for people with diabetes as for those without diabetes and advises adoption of international standards and local country guidelines aligned with the available resources in the health care delivery systems [32].

Factors to consider in the integration process

Integration of NCD and CD care requires harmonisation at regional, district and/or local levels, and consideration of the country-specific context in regards to physical and human resource capacity [32]. Moreover, all stakeholders should be on board, and the funding scheme should be synchronised. An effective harmonisation process reduces the need to organise new physical or human resources in the integrated care model; rather, it utilises the available resources to optimise holistic care delivery for tuberculosis and diabetes co-existence [21]. Considering the tuberculosis-diabetes double burden is disproportionately higher in LMICs, it is paramount that silo NCD and CD clinics are integrated and limited available resources are focused on integrated care to ensure the sustainability of integrated programs.

Traditionally, NCD and CD care programs have had different priorities, particularly in indicators and data collection tools used for monitoring and evaluation. This difference is significantly noted in LMICs, where donor partner priorities and metrics may heavily influence care models. Therefore, it is paramount to synchronise the tools for surveillance and monitoring for a sustained and effective integrated care program for tuberculosis and diabetes.

Healthcare facilities, including diabetes clinics, should have an infection control plan that includes tuberculosis-specific measures such as administrative and environmental protocols to reduce transmission of tuberculosis within this setting. These measures should adhere to existing recommendations in WHO’s International Guidelines for tuberculosis infection control.

Another component to consider is the use of community health workers to help implement tuberculosis and diabetes integrated care models. As many LMICs engage and scale up the incorporation of community health care workers [33], it is important to strengthen the capacity of these workers to take part in the tuberculosis and diabetes integrated package. Community health personnel can access underserved communities, help track patients, promote healthy behaviours, and connect people to formal healthcare facilities. These activities are vital to successful tuberculosis and diabetes prevention, diagnosis, and management.

Challenges of the integrated care model for NCD and CD clinics

The integrated care model for tuberculosis and diabetes is a complex one with a wide spectrum of challenges, including contained health care taskforce, patient factors, and infrastructure. At the patient level, important challenges include medication adherence, cost, and drug-drug interactions, as previously mentioned. Currently, the WHO recommends the same regimen to be prescribed to those with and without diabetes, with a close follow-up to monitor therapy response to ensure efficacy, safety, and identify complications from one or both conditions.

Regarding infrastructure, the main challenge is rooted in the funding scheme, particularly in LMICs that rely on foreign funds. Current funding models are fragmented with different sources and interests for different silos of NCD and CD care [34,35]. It remains a challenge to secure funding from a single source for an integrated care model. Historically, donors have their own specific targets, reporting requirements, and terms of reference for each disease. This poses a challenge for integrated models to have accountability and show impact within existing frameworks [34,35]. Furthermore, geopolitical changes further complicate the funding scheme through unexpected shifts outside of local control. Notably, the recent hiatus of the US Agency for International Development (USAID) and cuts to other foreign global health aid threaten to worsen public health threats such as HIV/AIDS, malaria and tuberculosis and erode progress toward improved tuberculosis and diabetes integrated care.

## Conclusions

Tuberculosis and diabetes are major global health concerns, with their prevalence projected to increase. The convergence and impact of these conditions are also expected to rise, making integrated care essential to avoid further strain on the already fragile healthcare delivery systems in LMICs. Integration of tuberculosis and diabetes care enhances bi-directional screening, promotes a holistic approach of treatment and management, and optimises outcomes for both diseases. Current challenges of combining NCD and CD clinics in LMIC resource-constrained settings include managing historically different funders, monitoring and evaluation systems, distribution of resources, and training of healthcare workers. Further implementation research is recommended to identify the most effective model for integrating clinics for tuberculosis and diabetes, as well as other NCDs and CDs. The tuberculosis and diabetes double burden of disease offers unique challenges in the present moment, but also new opportunities to transform the future of healthcare delivery globally. 
